# Co-delivery of sorafenib and metapristone encapsulated by CXCR4-targeted PLGA-PEG nanoparticles overcomes hepatocellular carcinoma resistance to sorafenib

**DOI:** 10.1186/s13046-019-1216-x

**Published:** 2019-05-31

**Authors:** Ning Zheng, Weiqun Liu, Bifei Li, Huifang Nie, Jian Liu, Yunlong Cheng, Jichuang Wang, Haiyan Dong, Lee Jia

**Affiliations:** 1 Cancer Metastasis Alert and Prevention Center, College of Chemistry, Fujian Provincial Key Laboratory of Cancer Metastasis Chemoprevention and Chemotherapy, Fuzhou University, Fuzhou, 350108 China; 2 Institute of Oceanography, Minjiang University, Fuzhou, 350108 Fujian China; 3 Fujian Key Laboratory for Translational Research in Cancer and Neurodegenerative Diseases, Institute for Translational Medicine, Fujian Medical University, Fuzhou, 350108 Fujian China

**Keywords:** Hepatocellular carcinoma, Sorafenib, Metapristone, SDF-1/CXCR4, PLGA-PEG, Combination therapy

## Abstract

**Background:**

Sorafenib is approved as a standard therapy for advanced hepatocellular carcinoma (HCC), but its clinical application is limited due to moderate therapeutic efficacy and high incidence of acquired resistance resulted from elevated levels of SDF-1/CXCR4 axis induced by prolonged sorafenib treatment. We previously demonstrated metapristone (RU486 metabolite) as a cancer metastatic chemopreventive agent targeting SDF-1/CXCR4 axis. Therefore, we hypothesized that combining sorafenib with metapristone could synergistically suppress cell proliferation, enhance anti-cancer activity and repress potential drug resistance.

**Methods:**

Changes in cellular CXCR4 expression by metapristone were analyzed by RT-PCR and western blotting. Effect of combining sorafenib with metapristone on cell viability was examined by MTT assay; combination index value was calculated to evaluate the synergistic effect of combined therapy. To overcome poor pharmacokinetics and reduce off-target toxicity, CXCR4-targeted nanoparticles (NPs) were developed to co-deliver sorafenib and metapristone into CXCR4-expressing HCC in vitro and in vivo; cell proliferation, colony formation and apoptosis assays were conducted; nude mice bearing HCC xenograft were used to examine effects of this therapeutic approach on HCC progression.

**Results:**

Here we showed metapristone significantly reduced CXCR4 expression in HCC. Combinatory chemotherapy of sorafenib with metapristone synergistically suppressed HCC proliferation and resistance. CXCR4-targeted PEGylated poly (lactic-co-glycolic acid) NPs conjugated with LFC131 (a peptide inhibitor of CXCR4), could deliver more sorafenib and metapristone into HCC via specific recognition and binding with transmembrane CXCR4, and resulted in the enhanced cytotoxicity, colony inhibition and apoptosis by regulating more Akt/ERK/p38 MAPK/caspase signaling pathways. Co-delivery of sorafenib with metapristone by the LFC131-conjugated NPs showed prolonged circulation and target accumulation at tumor sites, and thus suppressed tumor growth in a tumor xenograft model.

**Conclusions:**

In conclusion, co-delivery of sorafenib and metapristone via the CXCR4-targeted NPs displays a synergistic therapy against HCC. Our results suggest combinational treatment of chemotherapeutics offer an effective strategy for enhancing the therapeutic efficacy on carcinoma, and highlight the potential application of ligand-modified tumor-targeting nanocarriers in delivering drugs as a promising cancer therapeutic approach.

**Electronic supplementary material:**

The online version of this article (10.1186/s13046-019-1216-x) contains supplementary material, which is available to authorized users.

## Background

Hepatocellular carcinoma (HCC) is the sixth most prevalent malignancies and the third leading cause of cancer-related death around the world [1]. Systemic pharmacotherapy is the essential and final strategy for patients with advanced HCC, who are suffering from poor prognosis and limited therapeutic options [2]. In the clinic, sorafenib is the molecular targeted agent approved as the first-line systemic treatment for advanced HCC to improve overall survival of patients [3]. As an inhibitor of multiple tyrosine kinases, sorafenib exhibits the significant anti-cancer property via suppressing angiogenesis, inhibiting tumor cell proliferation and inducing apoptosis by blockade of the vascular endothelial growth factor receptor (VEGFR), platelet-derived growth factor receptor (PDGFR) and RAF/MEK/ERK pathway in HCC [4]. Unfortunately, only about 30% of HCC patients can benefit from sorafenib therapy, and most of them rapidly become resistant to sorafenib within 6 months, leading to the high incidence of HCC recurrence [3, 5]. Because of the high-level heterogeneity of individual response to sorafenib treatment in recent years, some researchers have investigated mechanisms of resistance to sorafenib [6], including activation of PI3K/Akt signaling pathway [7], epithelial-mesenchymal transition (EMT) [8] and intratumoral hypoxia induction [9]. There is an urgent need to develop a combinational chemotherapy of sorafenib with other reagents to enhance anti-tumor activity and overcome drug resistance.

Metapristone (RU42633) is the most predominant biologically active metabolite of mifepristone (RU486), and mifepristone is a synthetic steroid compound and widely used as an abortifacient for more than 30 years [10]. Our group has demonstrated the good safety and efficacy prolife of metapristone by showing its anti-proliferative and anti-metastatic effects on colorectal cancer [11], melanoma [12, 13], breast cancer [14], lung cancer [15, 16] and ovarian cancer [17]. Metapristone treatment intervened the EMT-related signaling pathway to realize breast cancer metastasis chemoprevention [14], suppressed non-small cell lung cancer (NSCLC) proliferation and metastasis by targeting RAS/RAF/MER/MAPK and EGFR-mediated PI3K/Akt pathways [15, 16]. In addition, we found metapristone was capable of greatly reducing expressions of CXCR4 to interrupt the SDF-1/CXCR4 chemokine axis and the related downstream signaling pathways, resulting in inhibition of cell proliferation, migration, invasion and functional adhesion of ovarian cancer [17].

The chemokine SDF-1 and its cognate receptor CXCR4 play multiple roles in HCC progression, including promoting angiogenesis, maintaining cancer growth, inducing EMT, facilitating invasion and dissemination, and aiding escape of immune surveillance [18]. Aberrant over-expression of CXCR4 is closely related to poor prognosis and aggressive tumor behavior of HCC [19, 20]. Chen and co-workers have demonstrated that prolonged sorafenib treatment heightens tumor hypoxia, and increases expressions of CXCR4 and SDF-1α in HCC [21, 22], which indicates that down-regulation of CXCR4 level or intervention of SDF-1/CXCR4 signaling pathway may overcome sorafenib resistance and evasion [23]. Based on our early study of CXCR4 expression decreased by metapristone in ovarian cancer [17], we hypothesized that the combinatory chemotherapy strategy using sorafenib in combination with metapristone could strengthen anti-cancer activity of sorafenib and suppress potential resistance to sorafenib in HCC.

Regardless of the potential clinical effects of combination therapies, poor water solution, unwanted off-target toxicity and poor pharmacokinetics of single agent restrict the application of combination approach in HCC patients in the clinic [3, 5]. Nanoparticulate drug delivery systems offer great promise in efficiently delivering such poorly soluble drugs to the specific tumor sites via enhanced permeability and retention (EPR) effect, resulting in decline of side effects and enhancement of therapeutic efficacy of drugs [24]. As a FDA-approved biocompatible and biodegradable polymer, poly (lactic-co-glycolic acid) (PLGA) is an efficient delivery carrier for hydrophobic chemotherapeutics [25]. PEGylated nanocarriers have been demonstrated to control drug release, promote drug stability in the circulation and decrease nonspecific uptake [26]. Ligand-conjugated nanoparticles (NPs) are designed and developed to endow nanocarriers with specific bind to biologically active molecules expressing on tumor cells, which further promotes active delivery of chemotherapeutics to tumor sites [27].

In the present study, we first investigated the effect of metapristone on CXCR4 expression and the anti-proliferative efficacy of sorafenib in combination with metapristone in HCC. In view of the association of CXCR4 expression with HCC progression, we designed CXCR4-targeted NPs to co-deliver sorafenib and metapristone to CXCR4-expressing HCC, with the purpose of enhancing the synergistic anti-tumor efficacy and reducing the off-target toxicity. The NPs were characterized in terms of particle size, charge and drug release profile. The specific recognition ability and anti-cancer effect of the CXCR4-targeted NPs were evaluated in the CXCR4-expressing HCC cells and tumor xenograft models.

## Methods

### Materials

LFC131 peptide (Tyr-Arg-Arg-Nal-Gly, MW 747.82) was synthesized and characterized by Sangon Biotech (Shanghai) Co., Ltd. PLGA (50/50) with terminal carboxylate groups (MW 15 kDa) and NH_2_-PEG-COOH (MW 3500) were purchased from Jinan Daigang Biomaterial Co., Ltd. (Jinan, China) and JenKem technology Co. Ltd. (Beijing, China), respectively. N-hydroxysuccinimide (NHS), 1-ethyl-3-(3-dimethylaminopropyl)-carbodiimide hydrochloride (EDC·HCl) and sorafenib (> 99%) were obtained from Aladdin Reagent Company (Shanghai, China). Coumarin 6 (C6) was purchase from Sigma-Aldrich (St. Louis, MO, USA). Metapristone was synthesized and purified (> 98%) in our laboratory as described previously [11].

### Preparation of drug-loaded PLGA-PEG NPs

PLGA-PEG-COOH copolymer was synthesized by conjugating COOH-PEG-NH_2_ to COOH-PLGA using an EDC/NHS technique as previously described by Cheng [28]. PLGA-PEG-COOH (100 mg) and sorafenib or metapristone (2 mg) were dissolved in tetrahydrofuran (THF, 10 ml; Sinopharm Chemical Reagent Co., Ltd., Shanghai, China) and stirred for 10 min as the oil phase. The mixture solution was added dropwise into deionized water (20 ml) under magnetic stirring. The NP suspension was stirred continuously overnight at room temperature to evaporate THF. Subsequently, sorafenib- or metapristone-loaded PLGA-PEG NPs (Sora-NPs or Meta-NPs) were purified by ultrafiltration (100,000 MWCO, Millipore Corporation, Bedford, MA) to remove un-encapsulated drugs. For preparation of drug-free NPs (Blank-NPs), drugs were expurgated from the above procedure. Coumarin 6-loaded NPs (C6-NPs) were synthesized as described above with addition of 0.15% (w/w) C6 for encapsulation.

### Conjugation of LFC131 peptide to drug-loaded NPs

Drug-loaded NPs (5 mg/ml) were incubated with EDC (200 mM) and NHS (100 mM) for 2 h under magnetic stirring. The NHS-activated NPs were washed repeatedly with deionized water and ultrafiltered to remove excess EDC and NHS, followed by reaction with LFC131 peptide for 12 h at room temperature with stirring continuously. The resulting LFC131-conjugated NPs were washed with phosphate buffered saline (PBS, PH 7.4) by ultrafiltration and collected at 4 °C for storage.

### Characterization of NPs

Zeta potential, particle size and polydispersity index (PDI) of NPs were determined by dynamic light scattering using a Zetasizer (Nano ZS, Malvern Instruments, Worcestershire, UK).

### Drug loading capacity (DLC) and drug encapsulation efficiency (DEE)

Amount of drugs encapsulated in different types of NPs was measured by UV-spectrophotometric method. Drug-loaded or LFC131-modified drug-loaded NPs were dissolved and disintegrated in THF to completely release the encapsulated drugs. Concentration of drugs was determined by a UV-vis spectrophotometer (UV2700, Shimadzu, Japan) at 267 nm (for sorafenib) or at 295 nm (for metapristone). Standard curve for drugs content calculation was obtained by detecting absorbance of predetermined concentrations of drugs at 267 nm for sorafenib and 295 nm for metapristone, respectively. The vehicle background was deducted by analyzing absorbance of Blank-NPs or LFC131-modified Blank-NPs (LFC-Blank-NPs) measured under the same condition.

### In vitro drug release

The in vitro drug release from NPs was investigated by dialysis method. Dialysis bags (8 ~ 14 kDa molecular weight cut-off; Beijing Dingguo Changsheng Biotechnology Co., Ltd., China) containing NP solution (2 ml) were immersed in 30 ml of PBS (0.01 M, pH 7.4 or 5.5) supplemented with 0.1% (v/v) Tween 80, with vibrating at 100 rpm at 37 °C in a constant temperature incubator shaker (Zhicheng Inc., Shanghai, China). At predetermined time interval (2, 4, 6, 8, 10, 24, 36, 48, 72, 96, 120, 144, 168, 192, 216 and 240 h), 1 ml of release medium was withdraw for measurement and replaced with the same volume of fresh medium. Amount of released drugs was determined by UV-spectrophotometric method as described above.

### Cell culture

HCC cell lines (HepG2, Huh7, and SMMC-7721 cells), which were kindly provided by Dr. Jichuang Wang (Institute for Translational Medicine, Fujian Medical University, Fuzhou, China), were maintained in Dulbecco’s modified Eagle’s medium (DMEM) supplemented with 10% (v/v) fetal bovine serum (FBS), 100 U/ml penicillin and 100 μg/ml streptomycin (all purchased from Hyclone Laboratories, Inc., Logan, UT, USA) at 37 °C in a humidified 5% CO_2_ incubator.

### In vitro cellular uptake study

The in vitro cellular uptake of NPs was performed by replacement of sorafenib and metapristone with C6, which was used as a tracer to emit fluorescence. Briefly, cells were seeded on coverslips and incubated overnight. The cells were cultured with different formulations containing C6 (3 μg/ml equivalent C6 concentration) at 37 °C in the dark. After treatment for 2 h, the cells were washed by PBS to remove unbound C6, fixed by 4% paraformaldehyde (Beijing Dingguo Changsheng Biotechnology Co., Ltd.) for 10 min, and counterstained with DAPI (Solarbio Science & Technology Co., Ltd., Beijing, China). Fluorescent images were captured to determine cellular uptake of C6 by a confocal microscope (Leica SP8, Leica, Solms, Germany).

To quantify cellular association of NPs by flow cytometric analysis, cells were plated in 6-well culture plates, incubated and treated as described above for various time points (1, 2 and 4 h). The cells were washed with PBS, harvested with 0.25% trypsin without EDTA, and suspended in PBS. Finally, fluorescence intensity distribution was analyzed by a flow cytometer (BD FACSAriaIII, BD Biosciences, San Jose, CA, USA).

The competitive cellular uptake assay was performed with cells pretreated with free LFC131 peptide (0.5 and 1 mg/ml) for 30 min prior to treatment with different C6-containing formations. After removal of peptide, the cells were processed and analyzed by using the same procedure as described above.

### Intracellular drug accumulation

The amount of sorafenib and metapristone retained by SMMC-7721 cells was determined by high performance liquid chromatography (HPLC; Waters e2695, Milford, USA) as previously described [29, 30]. Briefly, cells at a density of 5 × 10^5^ were plated in 6-well culture plates and incubated overnight. After incubation with indicated concentrations of drugs for 2 h, the cells were washed with ice-cold PBS and lysed with RIPA lysis buffer (Beyotime Biotechnology, Haimen, China) on ice. Protein concentration of the lysate was estimated by the BCA Protein Assay Kit (Beijing Dingguo Changsheng Biotechnology Co., Ltd.). To prepare samples for the HPLC analysis, acetonitrile (LiChrosolv, Merck, Germany) was added to precipitate proteins. The mixture was vortexed for 2 min and centrifuged at 10000 rpm for 10 min. Ethyl acetate (LiChrosolv) was mixed with the supernatant for extraction of drugs. After centrifugation at 12000 rpm for 10 min, the supernatant was collected completely and dried under nitrogen at 40 °C. The residue was dissolved in 120 μl methanol (LiChrosolv) and 20 μl of each sample was injected into the chromatographic system. Chromatographic separation was achieved on a Sunfire C18 column (4.6 mm × 150 mm, 5.0 μm; Waters). The composition of the mobile phase was 55% acetonitrile and 45% water containing 0.1% (v/v) formic acid (Anaqua Chemicals Supply Inc. Ltd., Houston, USA). The flow rate was 1 ml/min throughout the 15-min run. Chromatography was performed at 40 °C. Sorafenib and metapristone were monitored at a wavelength of 267 nm and 295 nm, respectively. Cellular drug accumulation was normalized to the total protein. A calibration curve was prepared by adding sorafenib or metapristone directly to the lysate from cells without treatment.

### MTT assay and synergy analysis

Effect of drugs on cell proliferation was examined by MTT assay. HCC cell lines were incubated in 96-well culture plates (5000 cells/well) for 12 h to adhere, and then treated with indicated concentrations of drugs for 24 h and 48 h, respectively. The cells were incubated with MTT (0.5 mg/ml, Genview, Gen-view scientific Inc., USA) solution at 37 °C for another 4 h, and formazan crystals were dissolved in dimethyl sulfoxide (DMSO). Finally, the absorbance was measured at 490 nm using a microplate reader (Tecan, Hombrechtikon, Switzerland). Synergistic effect of combination drugs was evaluated according to the Chou-Talalay method by Compusyn software (ComboSyn, Inc.). If combination index (CI) value of the two drugs is below 1, it can be assumed that they are synergistic [31].

### Colony formation assay

Cells in single-cell suspension were plated in 12-well culture plates at a density of 500 per well. After overnight attachment, the cells treated with indicated concentrations of drugs for 24 h. After incubation, the medium containing agents was removed and replaced with fresh medium. The cells were further cultured for 15 days at 37 °C until colonies were visible. The colonies were fixed with methanol (Sinopharm Chemical Reagent Co., Ltd.) and stained with 0.1% (w/v) crystal violet (Beijing Dingguo Changsheng Biotechnology Co., Ltd.). The numbers of colonies with more than 50 cells were counted under a light microscope (Zeiss). Triplicate wells were performed for each condition.

### Apoptosis assay

The drug-induced apoptosis was detected using an Annexin V-FITC/PI Apoptosis Detection Kit (Genview) according to the manufacturer’s instructions. Briefly, after incubation with indicated concentrations of drugs for 48 h, the cells were digested with 0.25% trypsin without EDTA, harvested with low-speed centrifugation, washed with PBS, and incubated with 5 μl Annexin V-FITC and 5 μl PI in 500 μl binding buffer for 10 min in the dark at room temperature. The stained cells were analyzed using a BD FACSAriaIII flow cytometer (BD Biosciences).

### Western blot analysis

The western blotting was performed as we described previously [12]. Briefly, after incubation with indicated concentrations of drugs for 24 h or 48 h, the cells were lysed in RIPA lysis buffer on ice. Equal samples of total protein were separated using SDS-PAGE and then transferred to PVDF membranes (Bio-Rad, Hercules, CA). The membranes were incubated with primary antibodies at 4 °C overnight, and HRP-conjugated secondary antibodies (Beyotime Biotechnology) for 2 h. Finally, the immunoreactive bands were visualized using an Efficient Chemiluminescence Kit (Genview) and photographed under a ChemiDoc XRS System (Bio-Rad). GAPDH was used as a loading control for western blotting.

The primary antibodies for western blotting were purchased from the following companies: anti-CXCR4 (sc-9046), anti-p-Akt1/2/3 (Ser473; sc-33,437), anti-p-ERK1/2 (Thr202/Tyr204; sc-16,982) and anti-GAPDH (sc-25,778) from Santa Cruz Biotechnology, Inc. (Santa Cruz, CA, USA); p-p38 MAPK (Thr180/Tyr182; #4511), anti-caspase-3 (#9665), anti-caspase-9 (#9502), anti-PARP (#9542), anti-p53 (#2527), anti-Bcl-2 (#1507) and anti-Bax (#5023) from Cell Signaling Technologies (Beverly, MA, USA).

### RT-PCR analysis

The RT-PCR assay was performed as we described previously [32]. Briefly, after incubation with indicated concentrations of drugs for 24 h, total RNA was isolated from the cells by a TRIzol reagent (Invitrogen, Carsbad, CA, USA). The real-time PCR was performed with a SYBR® Premix Ex Taq™ (TaKaRa, Dalian, China) on a CFX96 real-time PCR system (Bio-Rad) after cDNA synthesis with a PrimeScript™ RT reagent Kit (TaKaRa). Values were normalized to the house keeping gene GAPDH.

### Pharmacokinetic study

All animal studies were performed in accordance with animal protocol procedures approved by the Institutional Animal Care and Use Committee (IACUC) of Fuzhou University, which are consistent with AAALAS guidelines. All animals were monitored for abnormal behaviors to minimize animal pain and suffering. Animals were euthanized if excessive deterioration of animal health was noted.

Female BALB/c nude mice (purchased from Shanghai SLAC Laboratory Animal Co., Ltd., China) were randomly divided into three groups (*n* = 4 per group), and were respectively injected through the tail veins with free C6, C6-NPs and LFC131-modified coumarin 6-loaded NPs (LFC-C6-NPs) at a dose of 0.5 mg/kg C6. At 0, 0.5, 1, 2, 4 and 8 h post-administration, 100 μl blood was collected via the orbital veins and mixed with EDTA·2Na solution immediately. To prepare samples for analysis, 1.0 ml ethyl acetate (Sinopharm Chemical Reagent Co., Ltd.) was added to the blood sample. The mixture was vortexed and subsequently centrifuged at 12000 rpm for 10 min at 4 °C, and the supernatant organic phase was collected completely and dried under nitrogen at 40 °C in the dark. The residue was dissolved in 200 μl acetonitrile (Sinopharm Chemical Reagent Co., Ltd.), and transferred to black 96-well plates (Corning Costar, Cambridge, MA, USA). The fluorescence intensity was determined at excitation wavelength of 444 nm and emission wavelength of 505 nm using a microplate reader (Tecan). Concentration of C6 in blood samples was calculated based on a standard curve obtained by detecting fluorescence intensity of predetermined concentrations of C6 in blood.

### Tumor accumulation of NPs

Accumulation of NPs in tumors was performed in a tumor xenograft model. Female BALB/c nude mice were injected subcutaneously with human SMMC-7721 cells (6 × 10^6^ cells/0.1 ml/mouse). When the tumor volumes reached about 100 mm^3^, the nude mice were randomly assigned to three groups (*n* = 12 per group) and intravenously injected with free C6, C6-NPs and LFC-C6-NPs at a dose of 0.5 mg/kg C6. After administration for a determined time (1, 2 and 4 h), four mice in each group were euthanized and tumors were surgically removed. For determination content of C6, 50 mg tumor tissues were homogenized in 150 mg saline using a high-throughput tissue grinder (Scientz-48, Scientz, Ningbo, China). The tissue homogenate was treated with ethyl acetate and the C6 concentration in the supernatant was measured by fluorescence intensity analysis as described above. To further investigate the tumor uptake and distribution of NPs, the excised tumors were frozen in liquid nitrogen and sectioned at a thickness of 10 μm by a freezing microtome (Leica). The sections were visualized under a laser confocal microscope after counterstaining with DAPI.

### In vivo anti-tumor efficacy study

The therapeutic efficacy of different formulations was investigated in tumor-bearing nude mice which were established as described above. When the tumor volumes reached about 100 mm^3^, the nude mice were randomly assigned into seven groups (*n* = 5 per group). The mice of each group were administrated intravenously every other day for continuous 7 times with the different formulations as described in the following: (a) saline, (b) sorafenib (5 mg/kg), (c) metapristone (10 mg/kg), (d) combination of sorafenib (5 mg/kg) with metapristone (10 mg/kg), (e) LFC131-modified sorafenib-loaded NPs (LFC-Sora-NPs; sorafenib at a dose of 5 mg/kg), (f) LFC131-modified metapristone-loaded NPs (LFC-Meta-NPs; metapristone at a dose of 10 mg/kg) and (g) combination of LFC-Sora-NPs with LFC-Meta-NPs (LFC-Sora/Meta-NPs; sorafenib at a dose of 5 mg/kg and metapristone at a dose of 10 mg/kg). Tumor dimensions were monitored every other day with a vernier caliper, and tumor volumes were calculated according to the following formula: tumor volume = 0.5 × (length) × (width)^2^. After two days at the end of treatment, the mice were euthanized. The tumor tissues were excised, rinsed in saline, weighted and photographed.

### Statistics

All statistical analyses were performed by GraphPad Prism 5.0 software, if not otherwise stated. Results were shown as mean ± standard deviation (SD). Paired *t* test and one-way ANOVA was used to analyze comparisons between two groups and calculate differences among groups, respectively. Values of *P* < 0.05 were considered statistically significant.

## Results

### Metapristone enhanced anti-proliferative effect of sorafenib in HCC cells

**Fig. 1:**
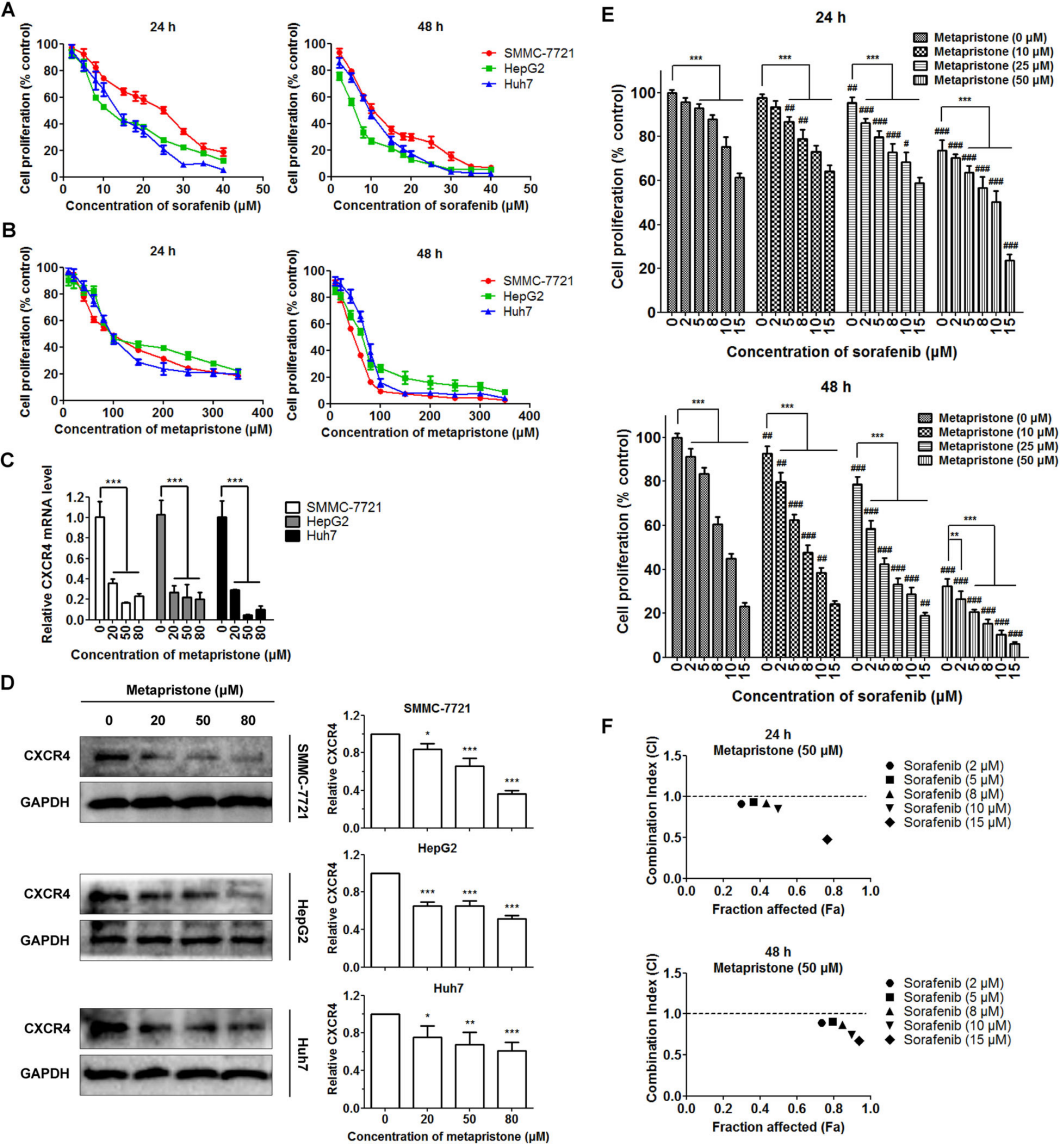
Effect of sorafenib, metapristone and their combination treatment on HCC cells proliferation. After treatment for 24 h and 48 h, the cell viability of SMMC-7721, HepG2 and Huh7 cells was inhibited by sorafenib (**a**) and metapristone (**b**) in a dose-dependent manner. Data are expressed as the mean ± SD (*n* = 5). After 24-h treatment, metapristone induced a remarkable decline of CXCR4 at the mRNA level (**c**) and at the protein level (**d**). The data were shown by western blot images (left panels) and quantitative analysis (right panels). Data are expressed as the mean ± SD (*n* = 3). *P < 0.05, **P < 0.01, ***P < 0.001. **e** The combination treatment of sorafenib (0, 2, 5, 8, 10 and 15 μM) with metapristone (0, 10, 25 and 50 μM) enhanced anti-proliferative effect in SMMC-7721 cells, as compared with either drug alone. Data are expressed as the mean ± SD (*n* = 5). Compared with the metapristone monotherapy group, *P < 0.05, **P < 0.01, ***P < 0.001; Compared with the sorafenib monotherapy group, #*P* < 0.05, ##*P* < 0.01, ###*P* < 0.001. **f** The combined effect of sorafenib (2, 5, 8, 10 and 15 μM) and metapristone (50 μM) was determined by CI values, which was calculated from the fraction-affected value of each combination using Compusyn software. CI > 1, antagonism; CI = 1, additive effect; CI < 1, synergism. All experiments were repeated at least three times

To evaluate the response of HCC cell lines to sorafenib and metapristone, HepG2, Huh7 and SMMC-7721 cells were treated with various concentrations of sorafenib or metapristone for 24 h and 48 h. Sorafenib and metapristone both inhibited cell proliferation of the three HCC cell lines in a dose-dependent manner (Fig. 1a and b). The IC50s of single drug on HCC cell lines were shown in Table 1.

**Table 1:** IC50s of sorafenib and metapristone on HCC cell lines in vitro. Data are expressed as the mean ± SD (*n* = 3)

HCC cell lines	IC50 (μM)			
	Sorafenib		Metapristone	
	24H	48H	24H	48H
SMMC-7721	21.87 ± 5.27	10.06 ± 3.69	120.30 ± 21.07	47.24 ± 8.44
HepG2	10.46 ± 2.69	5.93 ± 2.25	118.49 ± 15.28	62.23 ± 9.81
Huh7	13.14 ± 0.63	10.91 ± 0.69	106.06 ± 10.27	69.27 ± 4.55

Chen et al. verified that prolonged sorafenib treatment induced increases in levels of CXCR4 and its ligand SDF-1α in HCC, resulting in enhancement of cancer cell survival and promotion of metastatic phenotypes in HCC [21, 22]. In the previous study, we found metapristone significantly down-regulated CXCR4 expression in ovarian cancer, leading to inhibition of tumor progression and metastasis [17]. To explore the possibility of combination therapy and the underlying molecular mechanism, we first focused on effects of metapristone on CXCR4 level in HCC. The result showed that metapristone treatment produced a remarkable decline of CXCR4 at the mRNA level (Fig. 1c) and at the protein level (Fig. 1d). Here, we chose SMMC-7721 cells with the highest level of CXCR4 among these HCC cell lines to perform other experiments (Additional file 1: Figure S1).

Next, we assessed the potency of combination treatment of sorafenib with metapristone (Sora/Meta). SMMC-7721 cells were treated with different concentrations of sorafenib (0, 2, 5, 8, 10 and 15 μM) and metapristone (0, 10, 25 and 50 μM) for 24 h and 48 h. Combining sorafenib with metapristone produced a significant increase in inhibition of cell proliferation, when compared with either drug alone (Fig. 1e). The CI values of each combination were calculated by Compusyn software to evaluate the enhanced cytotoxic effect. The results indicated that low concentrations of metapritone (10 and 25 μM) showed an additive or even somewhat synergistic effect with sorafenib on suppressing SMMC-7721 cell proliferation (CI values approached 1, Additional file 2: Figure S2). Notably, combination of sorafenib with high concentrations of metapritone (50 μM) exhibited a remarkably synergistic cytotoxicity (CI values below 1, Fig. 1f).

### Physiochemical characterization of NPs

The preparation and proposed structure of LFC-Sora-NPs and LFC-Meta-NPs was depicted in Fig. 7a. Firstly, PLGA-PEG-COOH was successfully synthesized by EDC/NHS method as previously reported [28], and the chemical structure was characterized and confirmed by ^1^H NMR (Additional file 3: Figure S3). Sora-NPs or Meta-NPs were self-assembled in aqueous solution, with encapsulation of sorafenib or metapristone in hydrophobic core by employing intermolecular hydrophobic interactions between drugs and PLGA-PEG-COOH. As shown in Table 2, encapsulation of sorafenib or metapristone in PLGA-PEG NPs caused the increase of average diameters as compared to Blank-NPs, but didn’t affect the surface charge of NPs. To endow NPs with cell recognition and targeting capacity to trigger synergistic anti-tumor action, LFC131 peptide was conjugated to the surface of NPs by covalent bonds. The decrease in negative charge and increase in diameter were observed following addition of LFC131 (Table 2), which indicated a successful surface conjugation of LFC131 peptide with NPs. LFC131-modified and non-modified NPs both displayed uniform dispersal with PDIs of less than 0.17. DEE of sorafenib and metapristone in non-modified NPs was 54.82% ± 6.46 and 63.31% ± 12.75%, and in LFC131-modified NPs was 52.17% ± 3.92 and 61.34% ± 19.40%, respectively. DLC was 1.09% ± 0.13 and 1.03% ± 0.07% for sorafenib, and 1.27% ± 0.27 and 1.23% ± 0.34% for metapristone in LFC131-unmodified and modified NPs, respectively. The results revealed that the content of sorafenib or metapristone in NPs remained unchanged following conjugation of LFC131.

**Table 2:** Characterization of NPs

	Size (nm)	Zeta (mV)	PDI	DEE (%)	DLC (%)
Blank-NPs	106.70 ± 1.72	−32.9 ± 1.23	0.104 ± 0.015	–	–
LFC-Blank-NPs	124.30 ± 2.22	−14.80 ± 2.06	0.120 ± 0.022	–	–
Sora-NPs	112.37 ± 2.89	−33.13 ± 1.17	0.106 ± 0.027	54.82 ± 6.46	1.09 ± 0.13
LFC-Sora-NPs	145.10 ± 2.08	−15.47 ± 2.44	0.113 ± 0.012	52.17 ± 3.92	1.03 ± 0.07
Meta-NPs	114.80 ± 2.70	−32.80 ± 2.40	0.135 ± 0.030	63.31 ± 12.75	1.27 ± 0.27
LFC-Meta-NPs	147.10 ± 0.92	−17.80 ± 1.41	0.123 ± 0.065	61.34 ± 19.40	1.23 ± 0.34

Data are expressed as the mean ± SD (*n* = 3)

### In vitro sustained release of drugs from NPs

**Fig. 2:**
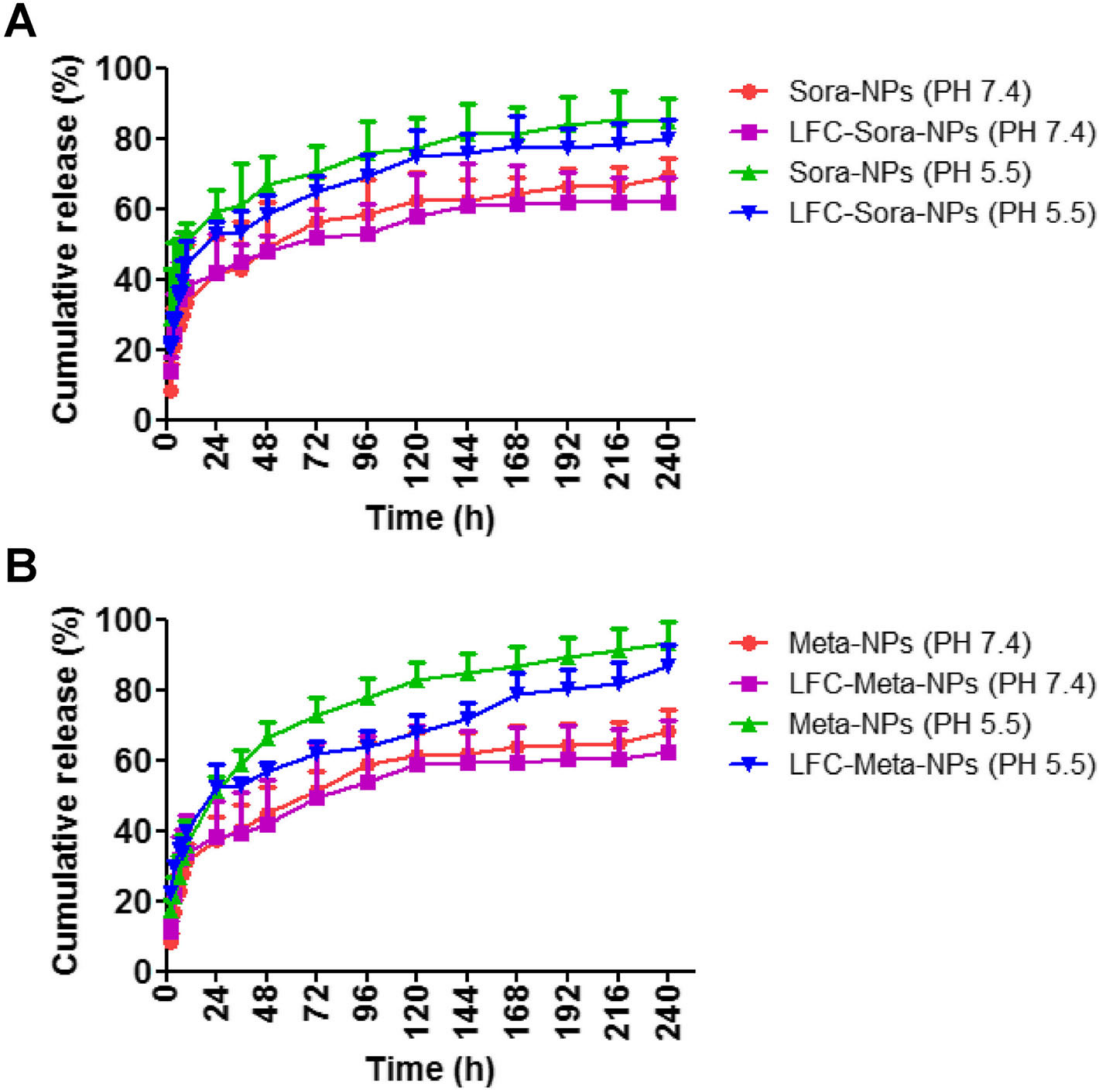
In vitro sustained release of drugs from PLGA-PEG NPs. The non-modified drug-loaded NPs and LFC131-conjugated drug-loaded NPs showed relatively fast drug release in the initial 24 h followed by sustained release patterns over a period of 192 h in PBS at pH 7.4 and pH 5.5. **a** Sora-NPs and LFC-Sora-NPs; **b** Meta-NPs and LFC-Meta-NPs. Data are expressed as the mean ± SD (*n* = 3)

The release profile of drugs from non-conjugated NPs and LFC131-conjugated NPs was investigated in PBS at pH 7.4 and pH 5.5 at 37 °C (Fig. 2). The two formulations showed relatively fast drug release in the initial 24 h followed by a sustained release pattern over a period of 192 h in both release medium. The drug release rate of LFC131-modified NPs was slightly lower than that of non-modified NPs in the same pH condition, which might be attributed to assembly of LFC131 on polymer layer to slow degradation of NP matrix. In addition, non-modified NPs and LFC131-modified NPs both exhibited a faster and stronger release pattern at pH 5.5 than that at pH 7.4. The release study suggested that PLGA-PEG NPs are capable of protecting effectively drugs in the systemic circulation and promoting drugs to rapidly release in tumor tissues with acidic microenvironments, which is beneficial for enhancing the anti-tumor effect of drugs.

### Conjugation of LFC131 enhanced in vitro cellular uptake of NPs

**Fig. 3:**
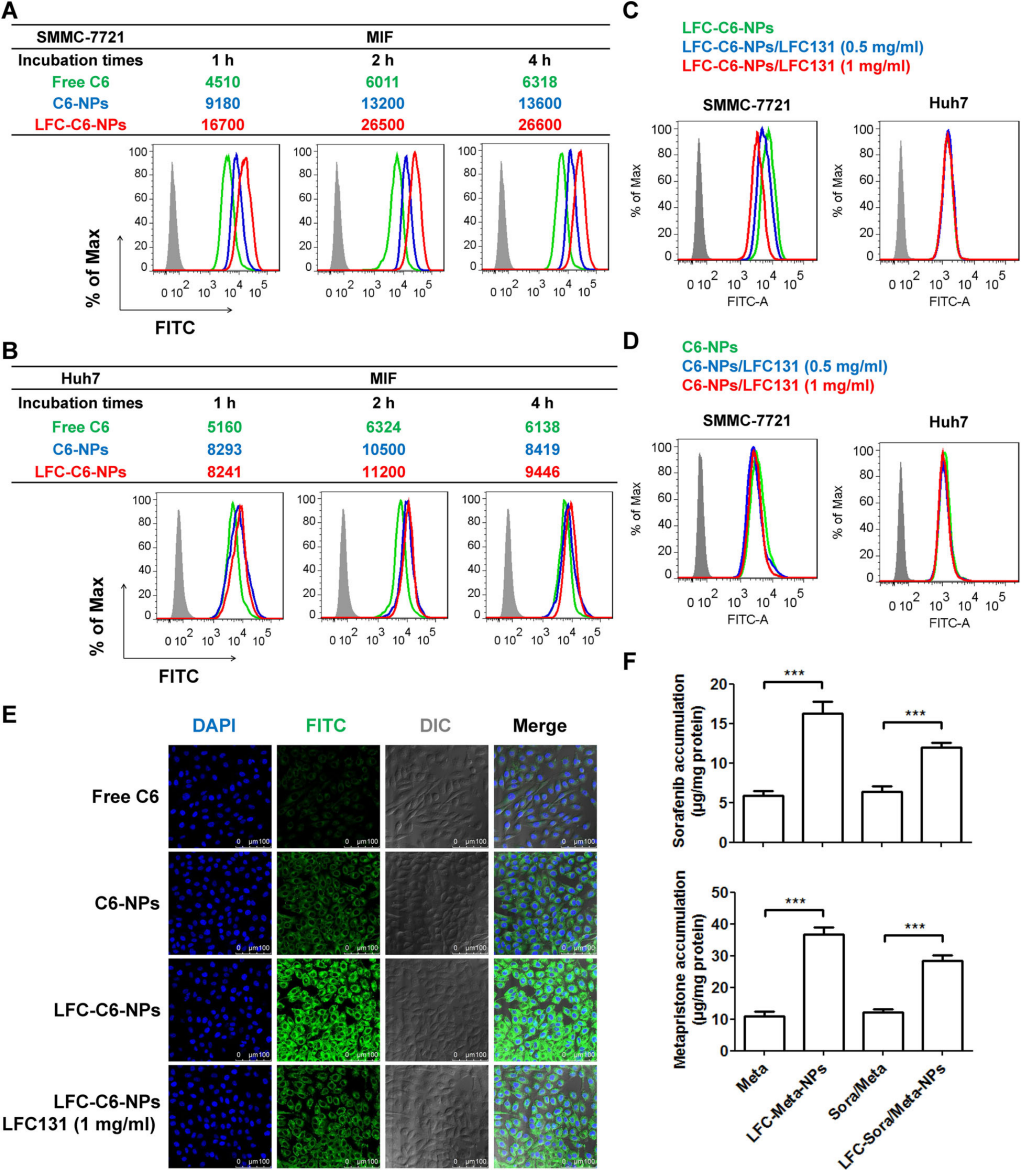
Modification of LFC131 enhanced cellular uptake of NPs in CXCR4-expressing HCC cells. Flow cytometric analysis of SMMC-7721 cells (**a**) and Huh7 cells (**b**) incubated with free C6, C6-NPs and LFC-C6-NPs for 1 h, 2 h and 4 h. The data were shown by flow cytometric images (lower panels) and quantitative analysis by the mean fluorescence intensity (MFI) for each sample (upper panels). Flow cytometric analysis of SMMC-7721 and Huh7 cells treated with LFC-C6-NPs (**c**) and C6-NPs (**d**) in the presence of free LFC-131 peptide (0.5 and 1 mg/ml). **e** Confocal microscopy images of intracellular C6 distribution in SMMC-7721 cells after incubation with the above formulations for 2 h. C6 (green) was used as a fluorescent probe; the nucleus of cells was stained with DAPI (blue). **f** HPLC analysis of intracellular accumulation of sorafenib and metapristone in SMMC-7721 cells after exposure to different formulations (sorafenib at a concentration of 20 μM; metapristone at a concentration of 100 μM) for 2 h. Data are expressed as the mean ± SD (*n* = 3). ***P < 0.001. All experiments were repeated at least three times

To measure whether modification of LFC131 promoted the cellular uptake of drugs, C6 was used as a fluorescent probe to be loaded in NPs. In the previous study, we found SMMC-7721 cells with higher level of CXCR4 than Huh7 cells by western blotting and flow cytometry (Additional file 1: Figure S1). We first executed flow cytometric analysis to quantitatively evaluate the uptake of free C6, C6-NPs and LFC-C6-NPs in SMMC-7721 cells and Huh7 cells after incubation for 1 h, 2 h and 4 h. In SMMC-7721 cells, the uptake of LFC-C6-NPs was obviously greater than that of C6-NPs and free C6 (Fig. 3a). LFC-C6-NPs delivered 2.2 times more C6 into SMMC-7721 cells as compared with C6-NPs, and 4.4 times more C6 than that of free C6 after 2-h treatment. After prolonging the period of incubation to 4 h, there was no longer increase in the cellular fluorescence intensity due to saturation of cellular uptake. In Huh7 cells, the uptake of C6 by delivery of C6-NPs and LFC-C6-NPs was much higher than that of free C6 treatment (Fig. 3b). However, the difference in cellular uptake between C6-NPs and LFC-C6-NPs was not pronounced. That was expected because of rare expression of CXCR4 in Huh7 cells. Furthermore, addition of free LFC131 peptide in SMMC-7721 cells significantly decreased the uptake of LFC-C6-NPs in a concentration-dependent manner (Fig. 3c), but did not affect the uptake of C6-NPs (Fig. 3d), which was attributed to competitive binding of free LFC131 peptide to CXCR4 receptor. In addition, the pretreatment of free LFC131 did not exert an influence on the uptake of C6-NPs and LFC-C6-NPs in Huh7 cells (Fig. 3c and d).

Next, confocal fluorescence images were used to further investigate internalization and intracellular distribution of NPs in SMMC-7721 cells after incubation for 2 h. As showed in Fig. 3e, the intracellular uptake of LFC-C6-NPs was significantly higher than that of C6-NPs and free C6, and greatly reduced in the presence of free LFC131peptide, which were consistent with the above flow cytometric analysis. In addition, we performed HPLC to detect intracellular accumulation of sorafenib and metapristone in SMMC-7721 cells after exposure to different formulations for 2 h (Fig. 3f). The higher intracellular levels of drugs were observed in LFC131-modified NPs-treated cells as compared with the cells treated with free drugs. The data suggested that PLGA-PEG NPs effectively promote intracellular drug delivery, and this delivery can be further improved by modification of NPs with CXCR4-targeting LFC131 peptide.

### Drug-loaded NPs enhanced anti-proliferative effect and promoted cell apoptosis

**Fig. 4:**
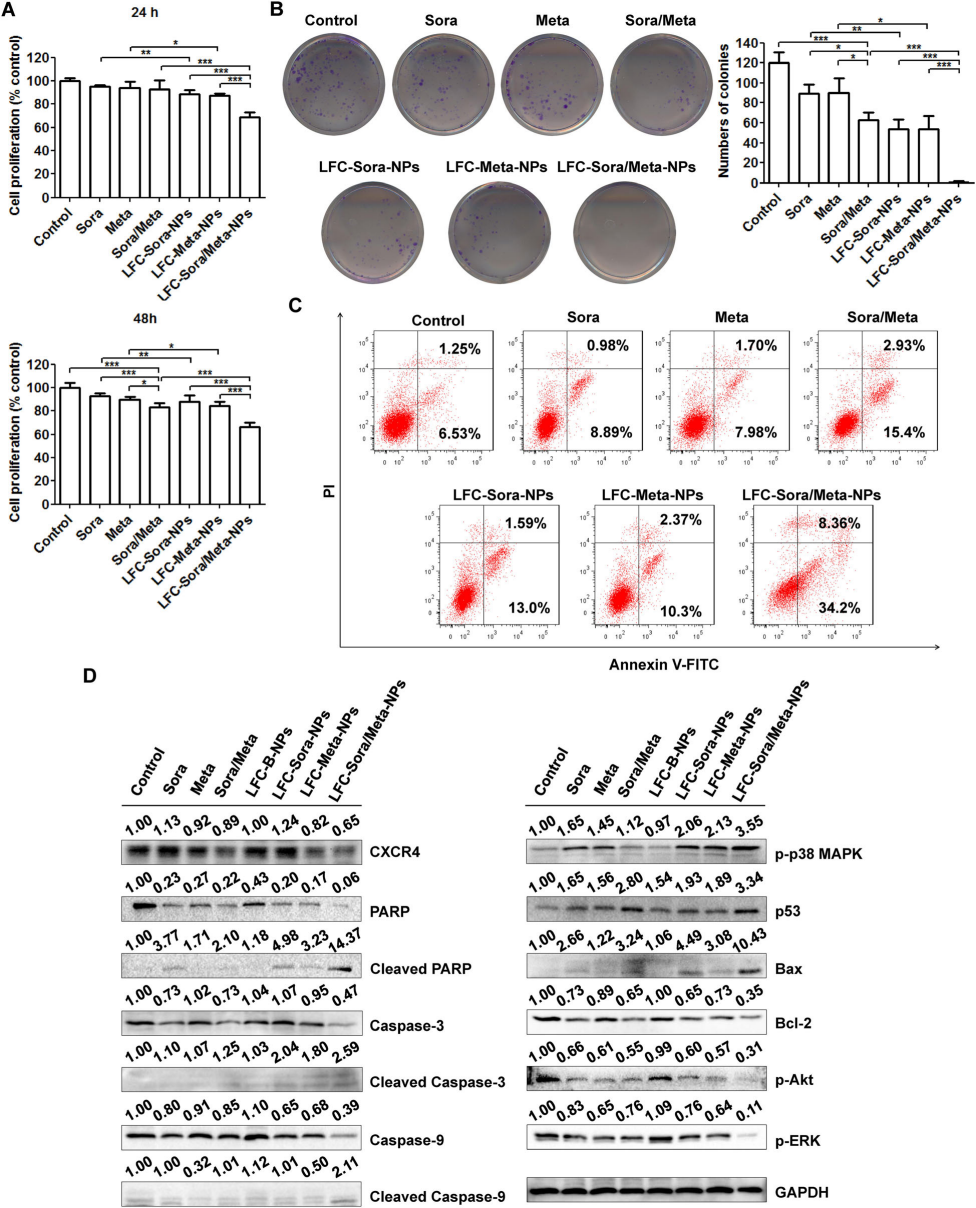
Co-delivery of sorafenib and metapristone by LFC131-modified NPs enhanced anti-proliferative activity and promoted cell apoptosis. **a** The in vitro anti-proliferative ability of different formulations in SMMC-7721 cells was determined by MTT assay after 24-h and 48-h incubation. Data are expressed as the mean ± SD (*n* = 5). *P < 0.05, **P < 0.01, ***P < 0.001. **b** After 24-h incubation, the effect of different formulations on SMMC-7721 cells colony formation was evaluated by colony formation assay. The data were shown by representative photographs (left panels) and quantitative analysis of colony numbers for each sample (right panels). Data are expressed as the mean ± SD (*n* = 3). *P < 0.05, **P < 0.01, ***P < 0.001. **c** After incubation with different formulation for 48 h, the apoptosis was determined by Annexin V-FITC/PI staining and characterized by the proportion of cells in early and late apoptotic phase by flow cytometry analysis. **d** The western blot analysis showed LFC-Sora/Meta-NPs significantly induced the activation of caspase-3, caspase-9 and PAPR, and increased the expression levels of p-p38, p53 and Bax, concomitant with the reduction in expression levels of CXCR4, Bcl-2, p-Akt and p-ERK, following incubation for 48 h. All experiments were repeated at least three times

The in vitro anti-proliferative ability of different formulations in SMMC-7721 cells was determined by MTT assay after 24-h and 48-h incubation. There was no apparent anti-proliferative activity found in LFC-Blank-NPs as compared with the control (data not shown). As shown in Fig. 4a, the monotherapy and combined therapy of sorafenib (2 μM) and metapristone (10 μM) at a low concentration did not show significant cytotoxicity in SMCC-7721 cells after a short period of incubation (24 h). However, the anti-proliferative effect of single agent was greatly improved by LFC131-modified NPs, which was attributed to more efficient delivery of drugs to SMMC-7721 cells. After prolonging the period of incubation (48 h), combination of sorafenib with metapristone exhibited an obviously synergistic cytotoxicity as compared with the monotherapy. Meanwhile, the superior cytotoxicity of LFC131-modified drug-loaded NPs was also observed following 48-h treatment. Moreover, LFC-Sora/Meta-NPs therapy exerted the strongest anti-proliferative activity as compared with other formulations after incubation periods of 24 h and 48 h. The similar inhibitory effect of different formulations on cell viability was observed in the colony formation assay (Fig. 4b). The numbers of colonies in treatment groups were significantly less than the control group, and LFC-Sora/Meta-NPs treatment almost completely suppressed the colony formation.

The apoptosis induction by different formulations in SMMC-7721 cells was determined by Annexin V-FITC/PI staining and characterized by the number of cells in early and late apoptotic phase (Fig. 4c). After combined therapy of sorafenib and metapristone for 48 h, the percentage of apoptosis was 18.33%, which had a significant improvement in apoptosis induction of the monotherapy of sorafenib (9.87%) and metapristone (9.68%). LFC131-modified drug-loaded NPs further augmented the number of apoptotic cells to 14.59% (for LFC-Sora-NPs) and 12.67% (for LFC-Meta-NPs). Consistent with the anti-proliferative activity, the treatment of LFC-Sora/Meta-NPs caused the highest proportion of apoptotic cells (42.56%) among the medication groups.

Next, we further explored the underlying mechanism by which LFC-Sora/Meta-NPs suppressed cell viability and induced apoptosis in SMMC-7721 cells. Despite the significant CXCR4 up-regulation that were observed in the cells after treatment with LFC-Sora-NPs, the LFC-Sora/Meta-NPs-treated cells showed the minimum levels of CXCR4 as compared to the control and the other medication groups (Fig. 4d). In addition, the western blot analysis showed LFC-Sora/Meta-NPs significantly induced activation of caspase-3, caspase-9 and PAPR, and increased expressions of p-p38 MAPK, p53 and Bax, concomitant with reduction in levels of Bcl-2, p-Akt and p-ERK (Fig. 4d). Together, the results suggested that sorafenib and metapristone co-delivered by LFC131-modified NPs can enhance anti-proliferative efficacy and promote tumor cell apoptosis via mediating Akt/ERK/p38 MAPK/caspase signaling pathways (Fig. 7b).

### Conjugation of LFC131 to NPs prolonged circulation time and enhanced tumor uptake of drugs

**Fig. 5:**
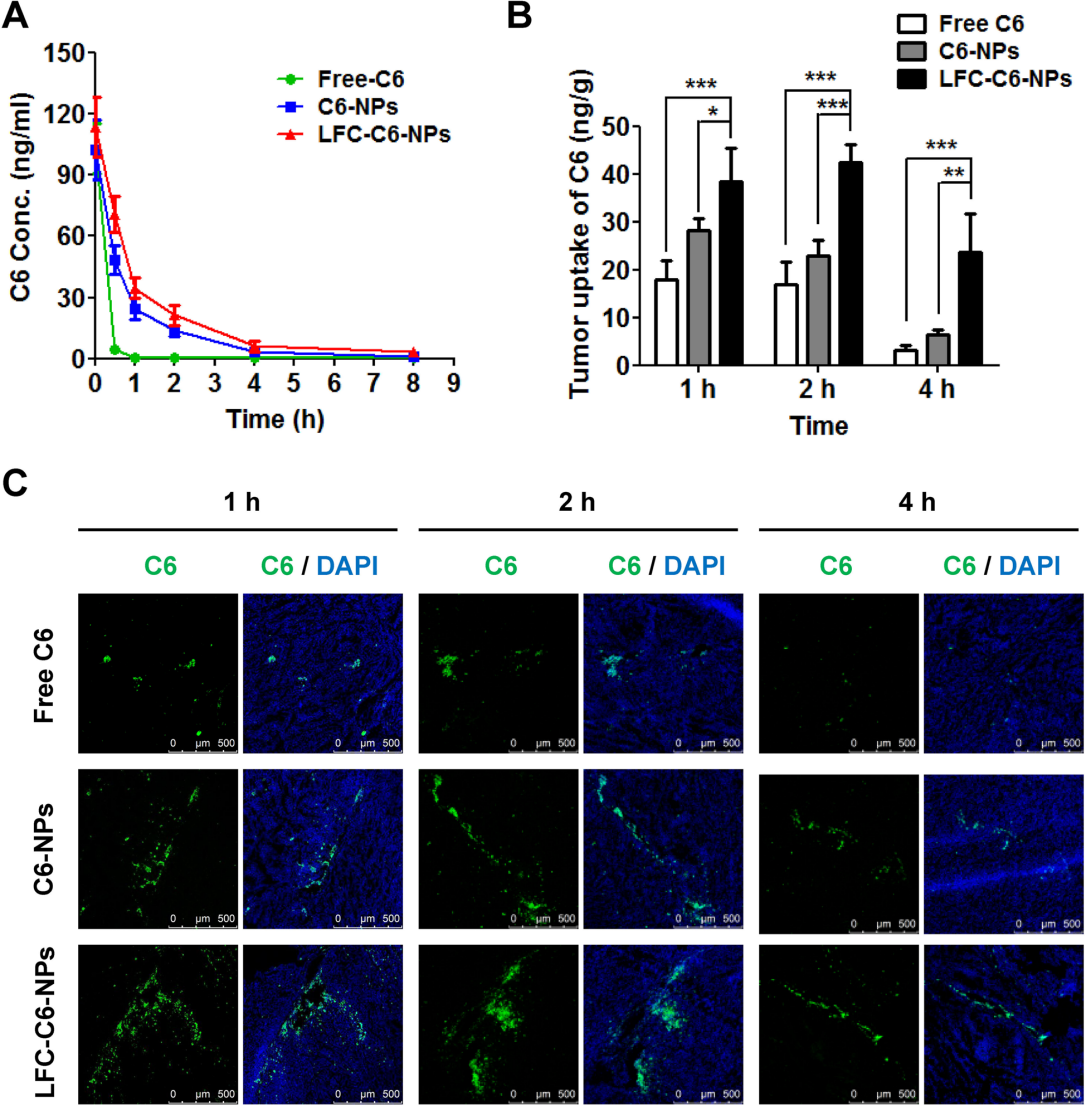
In vivo pharmacokinetics and tumor uptake of NPs. **a** Free C6 was rapidly cleared in the circulatory system, but C6-NPs and LFC-C6-NPs prolonged the circulation time of C6 in vivo. Data are expressed as the mean ± SD (*n* = 4). **b** LFC-C6-NPs exhibited much greater C6 delivery in tumor tissues than free C6 and C6-NPs. Data are expressed as the mean ± SD (*n* = 4). *P < 0.05, **P < 0.01, ***P < 0.001. **c** Following administration of free C6, C6-NPs and LFC-C6-NPs, the uptake and distribution of C6 (green) in tumor tissues was observed by confocal microscopy. The cell nuclei were counterstained with DAPI (blue)

The pharmacokinetic profile was performed in healthy BALB/c mice which were administrated intravenously with free C6, C6-NPs and LFC-C6-NPs at a dose of 0.5 mg/kg C6. As shown in Fig. 5a, C6 was cleared rapidly from the blood following administration of free C6. By contrast, C6-NPs and LFC-C6-NPs exhibited higher concentration of C6 and longer circulation time in the systemic circulation. Our data further indicated PLGA-PEG NPs could prolong circulation of drugs in vivo, which might be attributed to sustained release of encapsulated drugs and prevention of degradation in the circulatory system.

The distribution of NPs in tumor tissues was examined in a tumor xenograft model. LFC-C6-NPs exhibited much greater C6 delivery in tumor tissues than free C6 and C6-NPs (Fig. 5b). The result was further confirmed by confocal microscopy (Fig. 5c). A higher accumulation of C6 in tumors was observed following administration of LFC-C6-NPs as compared with that of free C6 and C6-NPs. The data suggested LFC131-modified NPs can effectively enhance tumor uptake of drugs.

### In vivo anti-tumor efficacy on nude mice bearing HCC xenograft

**Fig. 6:**
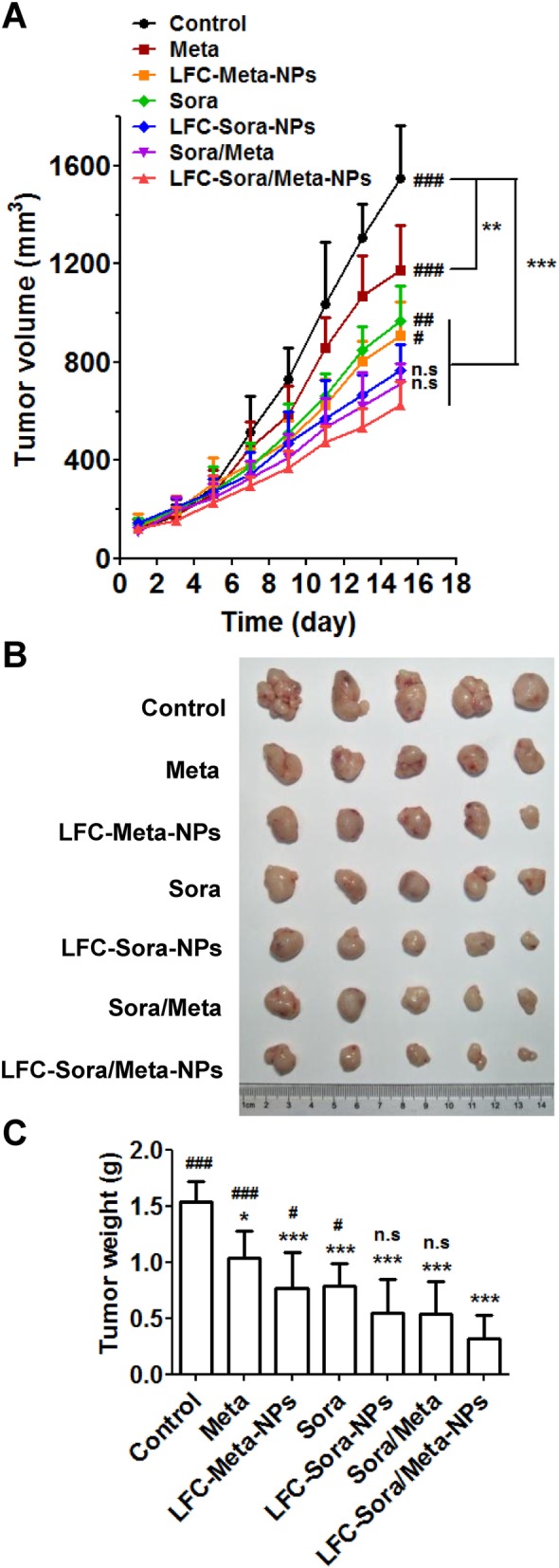
Evaluation of in vivo anti-tumor efficacy in the xenograft nude mouse model. **a** The tumor volumes were recorded every other day from the start of treatment until the end point. Photographs (**b**) and tumor weights (**c**) of the excised tumors from each group at the end point. Data are expressed as the mean ± SD (*n* = 5). Compared with the control group, *P < 0.05, **P < 0.01, ***P < 0.001; Compared with the LFC-Sora/Meta-NPs group, #*P* < 0.05, ##*P* < 0.01, ###*P* < 0.001

The in vivo therapeutic efficacy of different formulations was evaluated in nude mice bearing subcutaneous tumors induced by SMCC-7721 cells. The tumor growth inhibitory effect was initially observed on the 7th day after treatment and sustained until the experimental terminal in the following order: LFC-Sora/Meta-NPs > Sora/Meta ~ LFC-Sora-NPs > LFC-Meta-NPs ~ Sora > Meta > control (Fig. 6a). At the end point, the excised solid tumors were weighted (Fig. 6c) and photographed (Fig. 6b), and the result was highly consistent with the tumor volumes. The treatment of LFC-Sora/Meta-NPs resulted in a 79.76% reduction in tumor weights as compared with the control group, which was superior to that of other formulations (Fig. 6c). In addition, the average tumor inhibition rate of combination therapy of sorafenib and metapristone was 65.09%, which was 1.3-fold of sorafenib monotherapy and 2.0-fold of metapristone monotherapy. The LFC-Sora-NPs and LFC-Meta-NPs groups also respectively exhibited slightly higher inhibitory efficacy on tumor growth than their free drug monotherapy, owing to more efficient drug accumulation at tumor sites.

## Discussion

Recent reports suggest that sorafenib treatment can elicit hypoxic to activate HIF-1α in tumor cells and stromal cells in tumor microenvironment, leading to increases in expressions of CXCR4 and its ligand SDF-1α in HCC [21, 22]. Chen et al. indicated that inhibition of SDF-1α/CXCR4 chemokine axis in combination with sorafenib treatment restrained the polarization of tumor-accelerating microenvironment and enhanced therapeutic effect [21–23, 33]. Our group has demonstrated metapristone was a potential cancer metastatic chemopreventive agent derived from mifepristone [11–17]. In this study, we showed that metapristone treatment not only inhibited HCC cell proliferation (Fig. 1b), but also gave a rise to the remarkable decline in CXCR4 level in HCC cells (Fig. 1c and d). Moreover, metapristone exhibited an additive anti-proliferative effect at low concentrations and a synergistic cytotoxicity at high concentrations in combination with sorafenib, with the result of enhancing treatment efficacy of sorafenib (Fig. 1e and f).

**Fig. 7:**
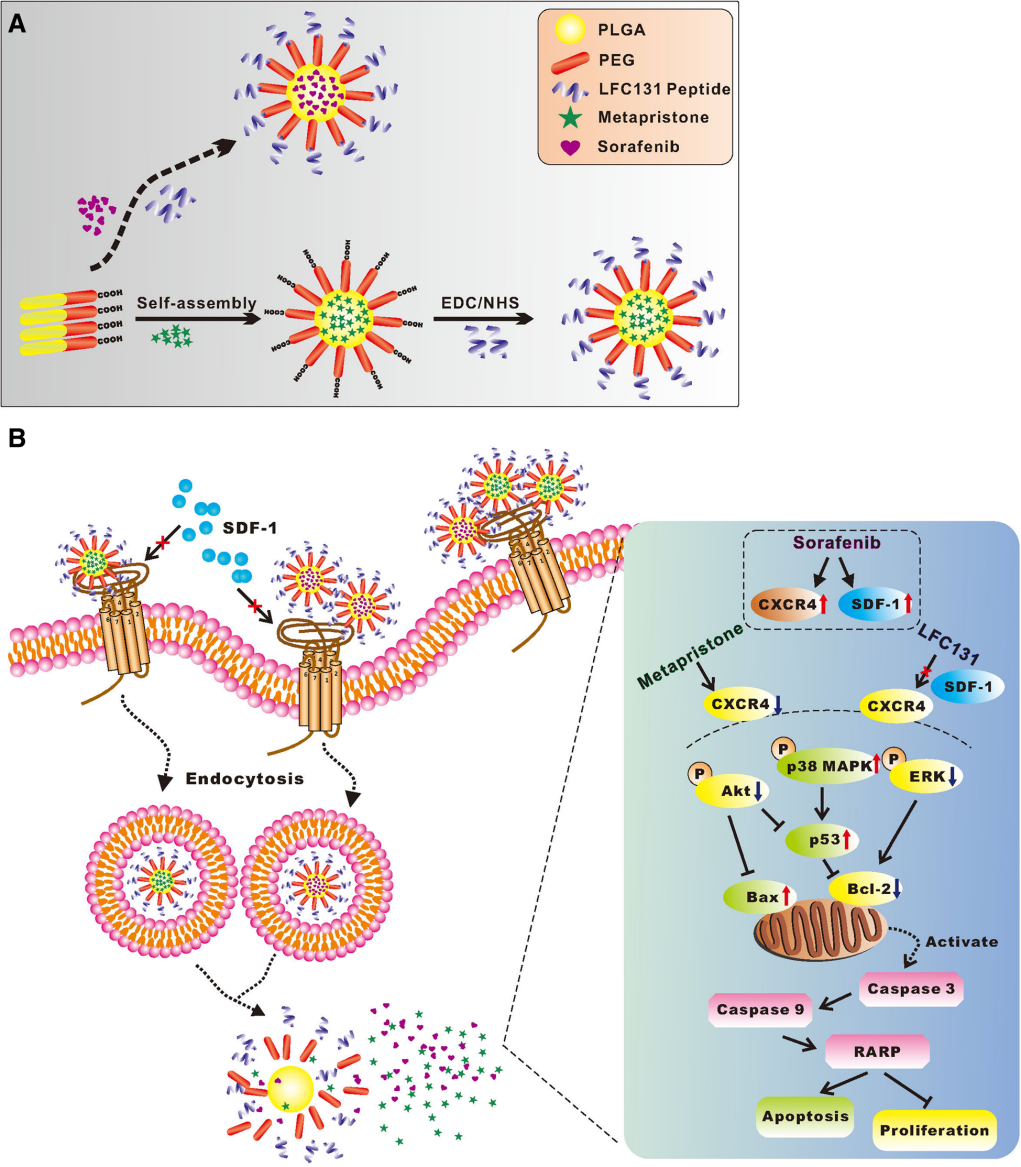
Schematic illustration of (**a**) the synthesis procedure of LFC-Meta-NPs and LFC-Sora-NPs, and (**b**) the mechanism by which combinational therapy of sorafenib with metapristone co-delivered by CXCR4-targeted PLGA-PEG NPs triggers synergistic anti-tumor effect on HCC. The decline of CXCR4 expression by metapristone and the blockade of CXCR4 function by LFC131 both greatly rescued the up-regulation and activation of SDF-1/CXCR4 upon prolonged sorafenib treatment, resulting in enhanced therapeutic effect of sorafenib and relief of potential resistance to sorafenib

Combining sorafenib with other chemotherapeutics has demonstrated to enhance anti-tumor efficacy and relieve acquired resistance to sorafenib, which provides a promising therapeutic strategy for treatment of HCC [34–36]. However, frequent adverse side effects and poor pharmacokinetics seriously limit clinical application of combinational therapy in patients with HCC [37–39]. Nanocarriers offer a safer and powerful treatment modality for cancer treatment through efficiently delivering therapeutic agents into targeted tumor tissues. In the study, PLGA-PEG, an amphiphilic copolymer, was employed for delivery of sorafenib and metapristone by self-assembly (Fig. 7a). These two hydrophobic drugs were assembled into the inner hydrophobic core of NPs via intermolecular hydrophobic interactions. The hydrophobic inner core was surrounded by hydrophilic outer shells, which provided a stable interface between the hydrophobic core and the aqueous environment [25]. Subsequently, the surface of PLGA-PEG NPs was modified with CXCR4 antagonist LFC131 peptide to achieve ligand-mediated targeting CXCR4-expressing HCC cells and tumors (Fig. 7a).

In this study, we employed the EDC/NHS technology to chemically conjugate LFC131 providing NH_2_ groups to surface of PLGA-PEG NPs offering COOH groups. This conjugation was confirmed by zeta potential and particle size (Table 2). The negative surface charge of PLGA-PEG NPs was attributed to existence of carboxyl modified PEG on the NPs surface [40]. The reduction in negative charge of NPs following modification with LFC131 was due to the decrease of carboxyl on the surface. The particle size of targeted NPs was larger than that of non-targeted NPs further confirming the conjugation of peptide. The mean diameters of peptide-conjugated NPs and non-conjugated NPs were both < 200 nm, which had favorable EPR effect to achieve passive tumor targeting [24]. In order to release drugs at a sustainable rate from PEGylated PLGA NPs and ameliorate its polydispersity, drug payload should be limited, unless using high molecular weight PLGA [28]. Cheng et al. pointed out that in our polymer system, NPs loading with about 1% drugs showed minimum polydispersity and might behave most predictably in vivo [28]. In our study, targeted NPs and non-targeted NPs both possessed a narrow size distribution (PDI < 0.17). In addition, their DLC and DEE were consistent with that of the similar polymer systems reported by others [40, 41]. All of these data corroborated success of formulation procedures and achievement of nice drug encapsulations. In consideration of DLC with only about 1% in our polymer system, we separately encapsulated these two hydrophobic drugs into two identical nanovehicles followed by combinatorial delivery.

In our nanoscale drug delivery system, the fast release of drugs from NPs at the initial stage might result from the diffusion of drugs which absorbed or were poor entrapped on surface of NP matrix (Fig. 2). The subsequent sustained release phase could be attributed to progressive degradation of NP matrix and gradual diffusion of drugs stably encapsulated in core of polymeric NPs [42]. The drug release rate of non-targeted NPs was slightly higher than that of CXCR4-targeted NPs, which might result from delaying matrix erosion and increasing particle size following LFC131 peptide conjugation [40]. The difference in the release behaviors between pH 7.4 and pH 5.5, indicated that the preferential release at tumor sites and the reduction of off-target cytotoxicity during circulation could be achieved by PLGA-PEG NPs.

The CXCR4 antagonists, such as T140, FC131, CTCE9908 and AMD3100, can effectively block functional CXCR4 to intervene SDF-1/CXCR4-mediated tumor progression and metastasis [43, 44]. Ligand conjugation in NPs carries dual biological activity through providing a targeting ligand for delivery of chemotherapeutics into malignant tumors as well as working as a blocker of signaling pathways [27]. The therapeutic strategies of CXCR4-targeted drug delivery system with modification of CXCR4 antagonist on NPs surface, have displayed superior anti-tumor effects as compared to that of passive approaches [23, 33, 45, 46]. LFC131, a liner type of FC131, shows efficient binding affinity for CXCR4 with possession of CXCR4 antagonistic activity [47]. LFC131 peptide modified on surface of NPs as a targeting ligand, has been reported to efficiently improve targeting ability of carriers, enhance therapeutic efficacy and decrease side effects in various cancer cells [48–50]. In the current study, LFC131-modified NPs showed higher intracellular uptake than non-modified NPs in CXCR4-expressing cells, and this superiority was weakened in the presence of excess LFC131, which suggested cellular association of LFC131-modified NPs is mediated by CXCR4-ligand specific interaction as well as EPR effect (Fig. 3).

Of note, via substantially facilitating cellular uptake, LFC131-modified drug delivery system effectively enhanced anti-proliferative activity, inhibited the formation of colonies, and elevated the level of apoptotic tumor cell death (Fig. 4). Meanwhile, co-delivery of sorafenib and metapristone by LFC131-modified NPs displayed the most superior anti-cancer effect among medication groups via mediating Akt/ERK/p38 MAPK/caspase signaling pathways (Fig. 4). In addition, LFC-Blank-NPs did not show obvious cytotoxicity in our experiment (data not shown), which was consistent with previous studies [23, 25, 40]. Our in vivo study demonstrated PLGA-PEG NPs successfully protected drugs from degradation and prolonged circulation time as compared with the single free drug administration (Fig. 5a). As expected, the conjugation of LFC131 peptide on the surface of PLGA-PEG NPs significantly promoted the delivery and accumulation of anti-cancer drugs at tumor sites (Fig. 5b and c), accompanied by the enhancement of anti-tumor activity in the xenograft nude mouse model (Fig. 6). Likewise, co-delivery of sorafenib and metapristone by LFC131-modified NPs possessed the strongest inhibitory effect on tumor growth among all chemotherapy groups (Fig. 6).

LFC131 peptide attached to nanocarriers possessed dual functions, serving as a ligand for targeted co-delivery of metapristone and sorafenib into malignant CXCR4-expressing HCC as well as a blocker for CXCR4. As a peptide inhibitor of CXCR4-ligand binding, LFC131 could prevent the effects of SDF-1 continuously up-regulated by sorafenib treatment, and sensitize HCC to sorafenib therapy via suppressing SDF-1/CXCR4-stimulated cancer cell proliferation, which was similar to AMD3100 reported by Chen [23, 33]. In the present study, combinational therapy of metapristone and sorafenib delivered by CXCR4-targeted PLGA-PEG NPs, was performed to synergistically inhibit HCC progression through reducing CXCR4 expression by metapristone and blocking interaction of SDF-1/CXCR4 by LFC131 peptide (Fig. 7b). These dual treatments could give rise to enhanced therapeutic effect and alleviative resistance to sorafenib.

## Conclusions

In summary, we found metapristone could inhibit cell proliferation and reduce CXCR4 expression in HCC cell lines. Combining sorafenib with metapristone triggered the synergistic anti-proliferative effect, and enhanced the chemotherapeutic effect on HCC. We prepared PLGA-PEG NPs that were modified with LFC131 peptide targeting CXCR4 to specifically and effectively co-deliver sorafenib and metapristone into CXCR4-expressing HCC, subsequently achieved the increase of cytotoxicity and the enhancement of apoptosis. The CXCR4-targeted drug delivery system showed uniform nanometer-particle size distribution, nice drug encapsulation, controlled drug release profile, persistent blood circulation, LFC131-receptor mediated recognition and preferential accumulation in tumors, which resulting in significantly enhancing the inhibition of tumor growth. The combined delivery of sorafenib and metapristone via CXCR4-targeted NPs exhibited superior anti-tumor efficacy to all of the other formulations. In this therapeutic strategy, the decline of CXCR4 expression by metapristone and the blockade of CXCR4 function by LFC131 both greatly rescued the up-regulation and activation of SDF-1/CXCR4 upon prolonged sorafenib treatment. Our results suggested combinational treatment of chemotherapeutics offer an effective strategy for enhancing the therapeutic efficacy on carcinoma, and highlighted the potential application of ligand-modified tumor-targeting nanocarriers in delivering drugs as a promising cancer therapeutic approach.

## Additional files

Additional file 1: **Figure S1.** CXCR4 expression in HCC cell lines. (DOCX 109 kb)

Additional file 2: **Figure S2.** CI values of combination treatment of sorafenib with metapristone. (DOCX 216 kb)

Additional file 3: **Figure S3.**^1^H NMR spectra of PLGA-PEG-COOH copolymer. (DOCX 93 kb)

## Abbreviations

C6: Coumarin 6
CI: Combination index
CXCR4: C-X-C chemokine receptor type 4
DAPI: 4′,6-diamidino-2-phenylindole
DEE: Drug encapsulation efficiency
DLC: Drug loading capacity
DMEM: Dulbecco’s modified Eagle’s medium
DMSO: Dimethyl sulfoxide
EDC·HCl: 1-ethyl-3-(3-dimethylaminopropyl)-carbodiimide hydrochloride
EMT: Epithelial-mesenchymal transition
EPR: Enhanced permeability and retention
FBS: Fetal bovine serum
GAPDH: Glyceraldehyde 3-phosphate dehydrogenase
HCC: Hepatocellular carcinoma
HPLC: High performance liquid chromatography
HRP: Horse radish peroxidase
MFI: Mean fluorescence intensity
NHS: N-hydroxysuccinimide
NP: Nanoparticle
NSCLC: Non-small cell lung cancer
PBS: Phosphate buffered saline
PDGFR: Platelet-derived growth factor receptor
PDI: Polydispersity index
PEG: Polyethylene glycol
PI: Propidium iodide
PLGA: Poly (lactic-co-glycolic acid)
PVDF: Polyvinylidene difluoride membrane
SDF-1: Stromal-derived factor-1
THF: Tetrahydrofuran
VEGFR: Vascular endothelial growth factor receptor

## References

[1] Torre LA, Bray F, Siegel RL, Ferlay J, Lortet-Tieulent J, Jemal A (2015). Global cancer statistics, 2012. CA Cancer J Clin.

[2] Bruix J, Gores GJ, Mazzaferro V (2014). Hepatocellular carcinoma: clinical frontiers and perspectives. Gut..

[3] Llovet JM, Ricci S, Mazzaferro V, Hilgard P, Gane E, Blanc JF, de Oliveira AC, Santoro A, Raoul JL, Forner A (2008). Sorafenib in advanced hepatocellular carcinoma. N Engl J Med.

[4] Raoul JL, Kudo M, Finn RS, Edeline J, Reig M, Galle PR (2018). Systemic therapy for intermediate and advanced hepatocellular carcinoma: Sorafenib and beyond. Cancer Treat Rev.

[5] Cheng AL, Kang YK, Chen Z, Tsao CJ, Qin S, Kim JS, Luo R, Feng J, Ye S, Yang TS (2009). Efficacy and safety of sorafenib in patients in the Asia-Pacific region with advanced hepatocellular carcinoma: a phase III randomised, double-blind, placebo-controlled trial. Lancet Oncol.

[6] Chen J, Jin R, Zhao J, Liu J, Ying H, Yan H, Zhou S, Liang Y, Huang D, Liang X (2015). Potential molecular, cellular and microenvironmental mechanism of sorafenib resistance in hepatocellular carcinoma. Cancer Lett.

[7] Chen KF, Chen HL, Tai WT, Feng WC, Hsu CH, Chen PJ, Cheng AL (2011). Activation of phosphatidylinositol 3-kinase/Akt signaling pathway mediates acquired resistance to sorafenib in hepatocellular carcinoma cells. J Pharmacol Exp Ther.

[8] Huang XY, Ke AW, Shi GM, Zhang X, Zhang C, Shi YH, Wang XY, Ding ZB, Xiao YS (2013). Yan J, et al: alphaB-crystallin complexes with 14-3-3zeta to induce epithelial-mesenchymal transition and resistance to sorafenib in hepatocellular carcinoma. Hepatology..

[9] Liang Y, Zheng T, Song R, Wang J, Yin D, Wang L, Liu H, Tian L, Fang X, Meng X (2013). Hypoxia-mediated sorafenib resistance can be overcome by EF24 through Von Hippel-Lindau tumor suppressor-dependent HIF-1alpha inhibition in hepatocellular carcinoma. Hepatology..

[10] Chen J, Wang J, Shao J, Gao Y, Xu J, Yu S, Liu Z, Jia L (2014). The unique pharmacological characteristics of mifepristone (RU486): from terminating pregnancy to preventing cancer metastasis. Med Res Rev.

[11] Wang J, Chen J, Wan L, Shao J, Lu Y, Zhu Y, Ou M, Yu S, Chen H, Jia L (2014). Synthesis, spectral characterization, and in vitro cellular activities of metapristone, a potential cancer metastatic chemopreventive agent derived from mifepristone (RU486). AAPS J.

[12] Wang J, Chen J, Zhu Y, Zheng N, Liu J, Xiao Y, Lu Y, Dong H, Xie J, Yu S (2016). In vitro and in vivo efficacy and safety evaluation of metapristone and mifepristone as cancer metastatic chemopreventive agents. Biomed Pharmacother.

[13] Zheng N, Chen J, Liu W, Wang J, Liu J, Jia L (2017). Metapristone (RU486 derivative) inhibits cell proliferation and migration as melanoma metastatic chemopreventive agent. Biomed Pharmacother.

[14] Yu S, Yan C, Yang X, He S, Liu J, Qin C, Huang C, Lu Y, Tian Z, Jia L (2016). Pharmacoproteomic analysis reveals that metapristone (RU486 metabolite) intervenes E-cadherin and vimentin to realize cancer metastasis chemoprevention. Sci Rep.

[15] Zheng G, Shen Z, Chen H, Liu J, Jiang K, Fan L, Jia L, Shao J (2017). Metapristone suppresses non-small cell lung cancer proliferation and metastasis via modulating RAS/RAF/MEK/MAPK signaling pathway. Biomed Pharmacother.

[16] Shao J, Zheng G, Chen H, Liu J, Xu A, Chen F, Li T, Lu Y, Xu J, Zheng N, Jia L (2017). Metapristone (RU486 metabolite) suppresses NSCLC by targeting EGFR-mediated PI3K/AKT pathway. Oncotarget..

[17] Zheng N, Chen J, Li T, Liu W, Liu J, Chen H, Wang J, Jia L (2017). Abortifacient metapristone (RU486 derivative) interrupts CXCL12/CXCR4 axis for ovarian metastatic chemoprevention. Mol Carcinog.

[18] Ghanem I, Riveiro ME, Paradis V, Faivre S, de Parga PM, Raymond E (2014). Insights on the CXCL12-CXCR4 axis in hepatocellular carcinoma carcinogenesis. Am J Transl Res.

[19] Liu H, Liu Y, Liu W, Zhang W, Xu J (2015). EZH2-mediated loss of miR-622 determines CXCR4 activation in hepatocellular carcinoma. Nat Commun.

[20] Xiang ZL, Zeng ZC, Tang ZY, Fan J, Zhuang PY, Liang Y, Tan YS, He J (2009). Chemokine receptor CXCR4 expression in hepatocellular carcinoma patients increases the risk of bone metastases and poor survival. BMC Cancer.

[21] Chen Y, Huang Y, Reiberger T, Duyverman AM, Huang P, Samuel R, Hiddingh L, Roberge S, Koppel C, Lauwers GY (2014). Differential effects of sorafenib on liver versus tumor fibrosis mediated by stromal-derived factor 1 alpha/C-X-C receptor type 4 axis and myeloid differentiation antigen-positive myeloid cell infiltration in mice. Hepatology..

[22] Chen Y, Ramjiawan RR, Reiberger T, Ng MR, Hato T, Huang Y, Ochiai H, Kitahara S, Unan EC, Reddy TP (2015). CXCR4 inhibition in tumor microenvironment facilitates anti-programmed death receptor-1 immunotherapy in sorafenib-treated hepatocellular carcinoma in mice. Hepatology..

[23] Gao DY, Lin Ts T, Sung YC, Liu YC, Chiang WH, Chang CC, Liu JY, Chen Y (2015). CXCR4-targeted lipid-coated PLGA nanoparticles deliver sorafenib and overcome acquired drug resistance in liver cancer. Biomaterials..

[24] Maeda H (2015). Toward a full understanding of the EPR effect in primary and metastatic tumors as well as issues related to its heterogeneity. Adv Drug Deliv Rev.

[25] Fornaguera C, Dols-Perez A, Caldero G, Garcia-Celma MJ, Camarasa J, Solans C (2015). PLGA nanoparticles prepared by nano-emulsion templating using low-energy methods as efficient nanocarriers for drug delivery across the blood-brain barrier. J Control Release.

[26] D'Souza AA, Shegokar R (2016). Polyethylene glycol (PEG): a versatile polymer for pharmaceutical applications. Expert Opin Drug Deliv.

[27] Zhao J, Mi Y, Liu Y, Feng SS (2012). Quantitative control of targeting effect of anticancer drugs formulated by ligand-conjugated nanoparticles of biodegradable copolymer blend. Biomaterials..

[28] Cheng J, Teply BA, Sherifi I, Sung J, Luther G, Gu FX, Levy-Nissenbaum E, Radovic-Moreno AF, Langer R, Farokhzad OC (2007). Formulation of functionalized PLGA-PEG nanoparticles for in vivo targeted drug delivery. Biomaterials..

[29] Blanchet B, Billemont B, Cramard J, Benichou AS, Chhun S, Harcouet L, Ropert S, Dauphin A, Goldwasser F, Tod M (2009). Validation of an HPLC-UV method for sorafenib determination in human plasma and application to cancer patients in routine clinical practice. J Pharm Biomed Anal.

[30] Wei D, Zhang H, Peng R, Huang C, Bai R (2017). ABCC2 (1249G > a) polymorphism implicates altered transport activity for sorafenib. Xenobiotica..

[31] Chou TC (2010). Drug combination studies and their synergy quantification using the Chou-Talalay method. Cancer Res.

[32] Zheng N, Chen J, Liu W, Liu J, Li T, Chen H, Wang J, Jia L (2017). Mifepristone inhibits ovarian cancer metastasis by intervening in SDF-1/CXCR4 chemokine axis. Oncotarget..

[33] Liu JY, Chiang T, Liu CH, Chern GG, Lin TT, Gao DY, Chen Y (2015). Delivery of siRNA using CXCR4-targeted nanoparticles modulates tumor microenvironment and achieves a potent antitumor response in liver Cancer. Mol Ther.

[34] Mao J, Yang H, Cui T, Pan P, Kabir N, Chen D, Ma J, Chen X, Chen Y, Yang Y (2018). Combined treatment with sorafenib and silibinin synergistically targets both HCC cells and cancer stem cells by enhanced inhibition of the phosphorylation of STAT3/ERK/AKT. Eur J Pharmacol.

[35] Xu J, Lin H, Li G, Sun Y, Shi L, Ma WL, Chen J, Cai X, Chang C (2017). Sorafenib with ASC-J9((R)) synergistically suppresses the HCC progression via altering the pSTAT3-CCL2/Bcl2 signals. Int J Cancer.

[36] Sun D, Liu H, Dai X, Zheng X, Yan J, Wei R, Fu X, Huang M, Shen A, Huang X (2017). Aspirin disrupts the mTOR-raptor complex and potentiates the anti-cancer activities of sorafenib via mTORC1 inhibition. Cancer Lett.

[37] Cai L, Xu G, Shi C, Guo D, Wang X, Luo J (2015). Telodendrimer nanocarrier for co-delivery of paclitaxel and cisplatin: a synergistic combination nanotherapy for ovarian cancer treatment. Biomaterials..

[38] Miao L, Guo S, Zhang J, Kim WY, Huang L (2014). Nanoparticles with precise Ratiometric co-loading and co-delivery of gemcitabine monophosphate and cisplatin for treatment of bladder Cancer. Adv Funct Mater.

[39] Sui J, Cui Y, Cai H, Bian S, Xu Z, Zhou L, Sun Y, Liang J, Fan Y, Zhang X (2017). Synergistic chemotherapeutic effect of sorafenib-loaded pullulan-dox conjugate nanoparticles against murine breast carcinoma. Nanoscale..

[40] Guo J, Gao X, Su L, Xia H, Gu G, Pang Z, Jiang X, Yao L, Chen J, Chen H (2011). Aptamer-functionalized PEG-PLGA nanoparticles for enhanced anti-glioma drug delivery. Biomaterials..

[41] Zhang KL, Zhou J, Zhou H, Wu Y, Liu R, Wang LL, Lin WW, Huang G, Yang HH (2017). Bioinspired "active" stealth magneto-Nanomicelles for Theranostics combining efficient MRI and enhanced drug delivery. ACS Appl Mater Interfaces.

[42] Acharya G, Shin CS, Vedantham K, McDermott M, Rish T, Hansen K, Fu Y, Park K (2010). A study of drug release from homogeneous PLGA microstructures. J Control Release.

[43] Scala S (2015). Molecular pathways: targeting the CXCR4-CXCL12 Axis--untapped potential in the tumor microenvironment. Clin Cancer Res.

[44] Debnath B, Xu S, Grande F, Garofalo A, Neamati N (2013). Small molecule inhibitors of CXCR4. Theranostics..

[45] Sung YC, Liu YC, Chao PH, Chang CC, Jin PR, Lin TT, Lin JA, Cheng HT, Wang J, Lai CP (2018). Combined delivery of sorafenib and a MEK inhibitor using CXCR4-targeted nanoparticles reduces hepatic fibrosis and prevents tumor development. Theranostics..

[46] Chen Y, Liu YC, Sung YC, Ramjiawan RR, Lin TT, Chang CC, Jeng KS, Chang CF, Liu CH, Gao DY (2017). Overcoming sorafenib evasion in hepatocellular carcinoma using CXCR4-targeted nanoparticles to co-deliver MEK-inhibitors. Sci Rep.

[47] Tsutsumi H, Tanaka T, Ohashi N, Masuno H, Tamamura H, Hiramatsu K, Araki T, Ueda S, Oishi S, Fujii N (2007). Therapeutic potential of the chemokine receptor CXCR4 antagonists as multifunctional agents. Biopolymers..

[48] Chittasupho C, Lirdprapamongkol K, Kewsuwan P, Sarisuta N (2014). Targeted delivery of doxorubicin to A549 lung cancer cells by CXCR4 antagonist conjugated PLGA nanoparticles. Eur J Pharm Biopharm.

[49] Di-Wen S, Pan GZ, Hao L, Zhang J, Xue QZ, Wang P, Yuan QZ (2016). Improved antitumor activity of epirubicin-loaded CXCR4-targeted polymeric nanoparticles in liver cancers. Int J Pharm.

[50] Chittasupho C, Anuchapreeda S, Sarisuta N (2017). CXCR4 targeted dendrimer for anti-cancer drug delivery and breast cancer cell migration inhibition. Eur J Pharm Biopharm.

