# Supplementary material for: Co-delivery of sorafenib and metapristone encapsulated by CXCR4-targeted PLGA-PEG nanoparticles overcomes hepatocellular carcinoma resistance to sorafenib
Source: J Exp Clin Cancer Res. 2019 May 31;38:232. doi: 10.1186/s13046-019-1216-x (PMC6544999; doi:10.1186/s13046-019-1216-x)
Supplement: Supplementary file 1 — Figure S1. CXCR4 expression in HCC cell lines. (DOCX 109 kb) [file 13046_2019_1216_MOESM1_ESM.docx]

**
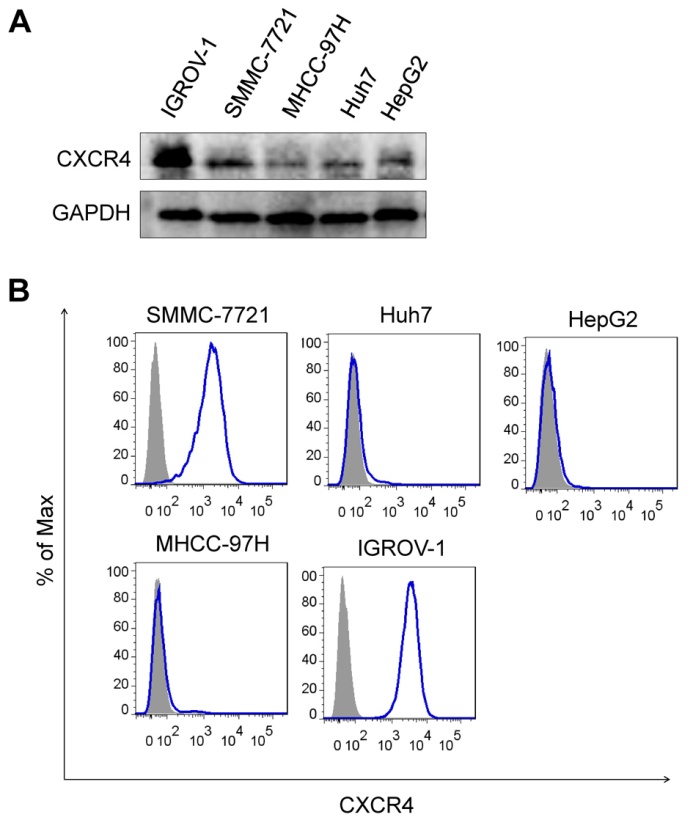
**

**Figure S1**. CXCR4 expression in HCC cell lines. Western blotting (A) and flow cytometry (B) were performed to evaluate the expression of CXCR4 in HCC cell lines, with ovarian cancer IGROV-1 cells as a positive control. All experiments were repeated at least three times.
