# Supplementary material for: Co-delivery of sorafenib and metapristone encapsulated by CXCR4-targeted PLGA-PEG nanoparticles overcomes hepatocellular carcinoma resistance to sorafenib
Source: J Exp Clin Cancer Res. 2019 May 31;38:232. doi: 10.1186/s13046-019-1216-x (PMC6544999; doi:10.1186/s13046-019-1216-x)
Supplement: Supplementary file 2 — Figure S2. CI values of combination treatment of sorafenib with metapristone. (DOCX 216 kb) [file 13046_2019_1216_MOESM2_ESM.docx]

**
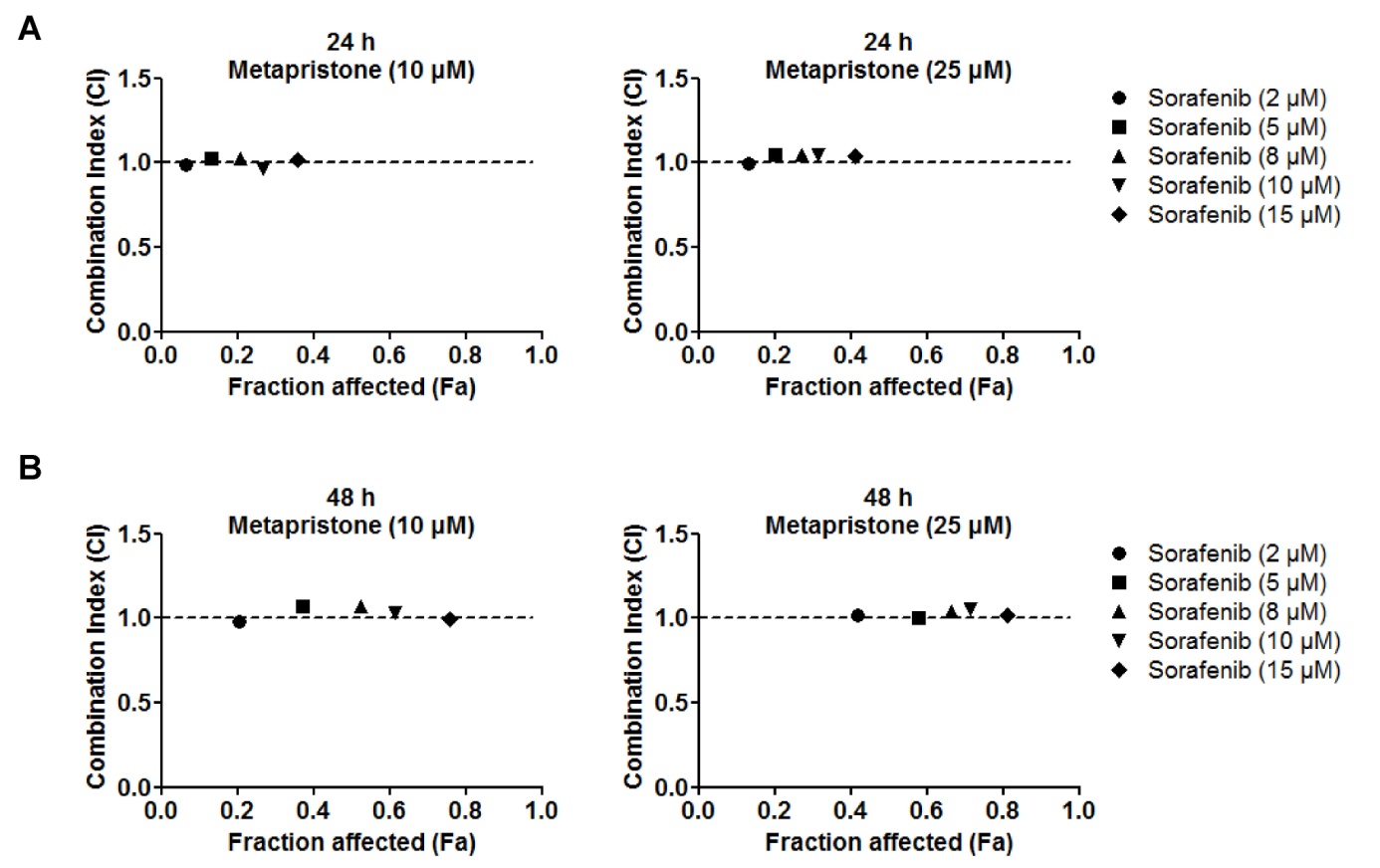
**

**Figure S2**. CI values of combination treatment of sorafenib with metapristone. The combined effect of sorafenib (2, 5, 8, 10 and 15 μM) and metapristone (10 μM, A; 25 μM, B) was determined by CI values, which was calculated from the fraction-affected value of each combination using Compusyn software. CI > 1, antagonism; CI = 1, additive effect; CI < 1, synergism. All experiments were repeated at least three times.
