# Supplementary material for: Co-delivery of sorafenib and metapristone encapsulated by CXCR4-targeted PLGA-PEG nanoparticles overcomes hepatocellular carcinoma resistance to sorafenib
Source: J Exp Clin Cancer Res. 2019 May 31;38:232. doi: 10.1186/s13046-019-1216-x (PMC6544999; doi:10.1186/s13046-019-1216-x)
Supplement: Supplementary file 3 — Figure S3.1H NMR spectra of PLGA-PEG-COOH copolymer. (DOCX 93 kb) [file 13046_2019_1216_MOESM3_ESM.docx]

**
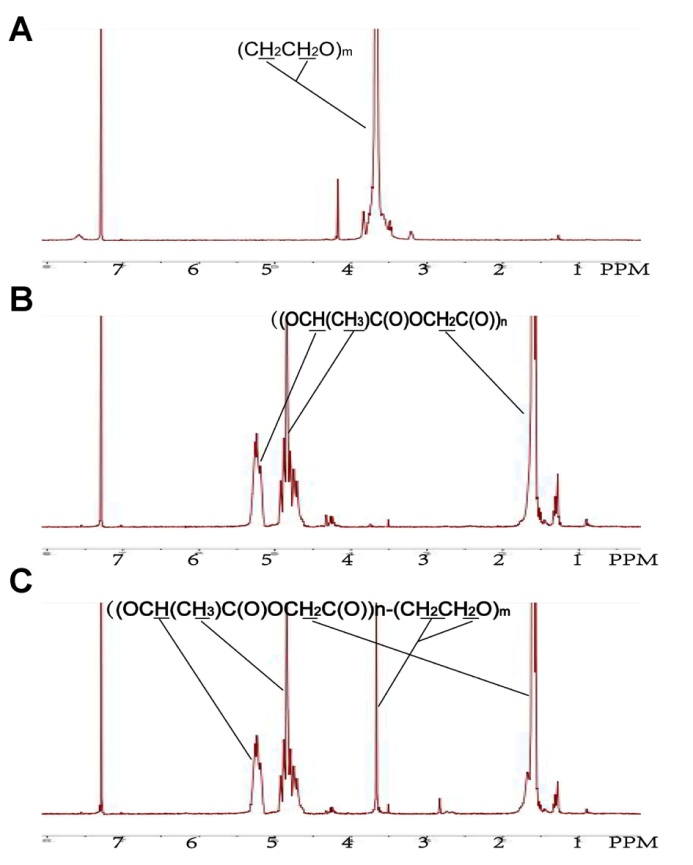
**

**Figure S3.** ^1^H NMR spectra of (A) PLGA-COOH, (B) NH2-PEG-COOH and (C) the synthesized PLGA-PEG-COOH copolymer dissolved in CDCl3. Characteristic peaks were marked in the graphs. The experiment was repeated at least three times.
